# KSHV-encoded vIL-6 collaborates with deregulated c-Myc to drive plasmablastic neoplasms in mice

**DOI:** 10.1038/bcj.2016.6

**Published:** 2016-02-26

**Authors:** T R Rosean, C J Holman, V S Tompkins, X Jing, M D Krasowski, S Rose-John, S Janz

**Affiliations:** 1 Department of Pathology, University of Iowa (UI) Carver College of Medicine, Iowa City, IA, USA; 2 Institute of Biochemistry, Christian-Albrechts-University, Kiel, Germany; 3 UI Holden Comprehensive Cancer Center, Iowa City, IA, USA

The mechanism by which Kaposi sarcoma (KS)-associated herpesvirus (KSHV), now recognized as human HV-8 (HHV-8), drives cancer is poorly understood.^1^ In addition to KS, the virus causes life-threatening pathologies of the mature B-lymphocyte lineage,^2^ including multi-centric Castleman's disease (MCD),^3^ diffuse large B-cell lymphoma evolving from MCD (MCD-DLBCL)^4^ and primary effusion lymphoma (PEL).^5^ These B-cell disorders are a significant public health concern because KSHV is frequently associated with HIV, which by itself increases the risk of developing B-lymphoma by ~2 orders of magnitude. In addition, the broad implementation of highly effective antiretroviral therapy has inadvertently caused a spike in lymphoma-related morbidity and mortality due to increased survival of HIV patients. Here, we took advantage of the laboratory mouse to enhance our understanding of KSHV-dependent pathophysiology in the B-lymphocyte lineage. Specifically, we evaluated the hypothesis that a KSHV-encoded cytokine, viral interleukin-6, hereafter called vIL-6, may be a driver of the transformation of B-lymphocytes to a malignant state. Our experimental strategy included the refinement of a recently developed C57BL/6 (B6) mouse model of constitutive, H2-K promoter-driven, transgenic (TG) vIL-6 expression^6^ by making two critical changes. First, we backcrossed the vIL6 transgene from the genetic background of B6 onto BALB/c (C), an inbred strain of mice that is hyper-susceptible to malignant plasma cell tumors, such as inflammation-dependent peritoneal plasmacytoma.^7^ Second, we intercrossed the newly generated C.vIL6 congenic mice with C.iMycΔEμ mice, a gene-insertion model of the chromosomal translocation T(12;15) that results in the deregulated expression of the cellular oncogene *Myc* in the B-cell lineage. We found that single-TG C.vIL6 mice are prone to a severe and sometimes fatal MCD-like disease, whereas double-TG C.vIL6iMyc mice invariably developed aggressive plasmablastic neoplasms that exhibited striking clinical and histopathological similarities to human PEL, plasmablastic lymphoma (PBL) and immunoglobulin (Ig)-producing, extramedullary plasmablastic plasma cell myeloma (PBM). The new findings will be briefly summarized below.

We previously reported that vIL6-TG B6 mice (i) exhibited vIL-6 serum levels comparable to those observed in KSHV-infected patients, (ii) contained elevated amounts of pSTAT3 in lymphoid tissues and (iii) developed, in dependency on mouse IL-6 (mIL-6), MCD-like changes with low genetic penetrance and late onset. However, malignant tumors were not seen.^6^ To assess the suitability of the vIL6 transgene for modeling KSHV-associated lymphoma, we first asked whether the transgene distorts the composition of the cell compartment in which malignant transformation is postulated to occur: mature B-lymphocytes. This is an important consideration for tumor studies using TG mice, because cancer may be the indirect outcome of developmental aberrations of the cell lineage that undergoes neoplastic transformation, the direct consequence of the oncogenic properties of the TG driver or a combination of both. Our analysis of the mature splenic B-cell compartment in vIL6-TG B6 mice demonstrated that the frequency of follicular, marginal zone and transitional B cells was comparable to that in age-matched normal B6 mice (Figure 1a). Non-immunized vIL6-TG mice did not exhibit spontaneous germinal centers (Figure 1b) or changes in the Ig isotype profile of B220^+^PNA^+^ B cells (Figure 1c) compared with controls. Likewise, flow cytometric analysis of vIL6-TG mice demonstrated no increase in CD138^+^B220^+^ plasmablasts or CD138^+^B220^−^ plasma cells in the spleen (Figure 1d) or other lymphoid tissues (not shown). These results agreed with a wholesale lack of lymph node enlargement and splenomegaly on necropsy of 10 randomly chosen mice ~8 months of age (not shown) and supported the view that the vIL6 transgene on the genetic background of B6 is a weak oncogene that renders this strain of mice impractical for cancer studies.

We transferred the vIL6 transgene to the genetic background of C (by means of introgressive backcrossing for 10 consecutive generations) to increase the efficacy with which enforced expression of vIL-6 drives malignant B-cell transformation (Figure 1e). The rationale for this approach was twofold. First, owing to a complex genetic trait that is determined by susceptibility genes in both the B-lymphocyte lineage (for example, *Cdkn2a* and *Mtor*) and the tumor microenvironment (for example, *Mndal*), C mice are highly susceptible to neoplasms of plasmablasts and plasma cells^8^—key constituents of KSHV-related B-cell disorders. Second, a previous study from our laboratory showed that the B6-to-C transfer of a human IL-6 transgene—controlled by a MHC-I promoter (H-2L^d^) that is very similar to the one driving vIL-6 expression (H-2K)—resulted in a dramatic phenotypic shift from benign hyperplasia (plasmacytosis) to malignant growth (plasmacytoma),^9^ thus providing a compelling precedent for the impact of the C genotype on vIL-6-dependent tumor formation. We generated a small cohort of C.vIL-6 N_10_ mice (*n*=10), observed them to 250 days of age and found that these mice developed MCD-like plasma cell proliferations faster (mean onset 219±14.6 days) and with higher incidence (7/10, 70%) than their B6 counterparts (7/25, 28%).^6^ Disease progression was evident by gross pathological changes such as splenomegaly (mean weight 337±220 mg) and general lymphadenopathy (Figure 1f) and by histopathologic changes including expansive accumulations of plasma cells in lymph nodes (Figure 1g), occurrence of abundant IgM^+^ plasma cells in inter-follicular splenic areas (Supplementary Figure 1a), increased numbers of megakaryocytes in medullary and extramedullary tissues (Supplementary Figure 1b) and aggregates of plasma cells at sites where cells of this sort normally do not occur (Supplementary Figure 1c). A single C.vIL-6 mouse developed a neoplasm of plasmablasts that resembled human extracavitary PEL/PBL (not shown). Serum protein electrophoresis of 6 C.vIL-6 mice 215 days of age uncovered polyclonal hyper-gammaglobulinemia and monoclonal extragradients (M-spikes) in three and two cases, respectively (Figure 1h, Supplementary Figure 2a, left). These findings demonstrated the amplification effect of C alleles on the oncogenic potency of vIL-6 in plasmablasts and plasma cells and indicated that the C.vIL6 model of human MCD (almost universally fatal if untreated^10^) is superior to the parental B6 model.

Recent approaches to improve the treatment of KSHV-associated B-cell disorders have focused on MYC (v-myc myelocytomatosis viral oncogene homolog) as a molecular target.^11^ MYC is also involved in the natural history of MCD-DLBCL and PEL; for example, by regulating KSHV's latent and lytic life cycles.^12^ On this backdrop, we decided to investigate whether vIL-6 collaborates with deregulated Myc in lymphoma development in mice. We generated a pilot cohort of double-TG C.vIL6iMyc mice (*n*=7) (Figure 2a) and determined their tumor-free survival. Tumor development was fully penetrant (100% tumor incidence) and tumor onset was short (140 days median) and predictable (110–160 days range; Figure 2b). Histopathological examination showed predominantly plasmablastic neoplasms that recapitulated many features of human PBL and/or extramedullary PBM (Figure 2c, top), although some areas contained plasma cell neoplasms similar to the extensive sheets of aberrant plasma cells seen in MCD-bearing C.vIL-6 mice (Figure 2c, center). The malignant plasma cells were readily detected by flow cytometry using antibody to CD138 (Figure 2d). Serum samples of four out of four tumor-bearing mice contained massive M-spikes that caused up to fivefold drops in albumin-to-globulin ratios (Figure 2e; Supplementary Figure 2a, right). Of note, three out of seven mice demonstrated tumor cell-laden cavitary effusions that resembled human PEL (Figure 2f). These tumors were as highly proliferative as the PBL-like neoplasm depicted in Figure 2g, using Ki67 immunoreactivity as measurement tool (up to 80% Ki67^+^ cells, not shown). We concluded that strain C.vIL6iMyc is prone to a spectrum of neoplasms that mimic features of human KSHV-associated malignancies including PEL. Furthermore, the iMyc transgene, which accelerated vIL6-dependent disease, may serve as surrogate of human MYC for preclinical studies on MYC-targeted therapies of KSHV-associated lymphoma.

The principal advance described in this Letter is improved preclinical modeling of vIL6-dependent pathophysiologies in the mature B-cell lineage. Strain C.vIL6 and C.vIL6iMyc mice may be useful for translational studies on new approaches to treat and prevent KSHV-associated, vIL-6-driven B-cell disorders more effectively. For example, clinical observations indicating that human IL-6 plays an important role in flares of KSHV-associated MCD^10^ may be corroborated by the assessment of vIL-6 TG mice that are either proficient for mouse IL-6 (mIL-6^+^) or deficient of it (mIL-6^−^). The latter mice can be readily generated by transferring the vIL6 transgene to C mice that are homozygous for a null allele of mIL-6.^6, 13^ Furthermore, adoptive transfer of vIL6iMyc-TG B cells to mIL-6^+^ or mIL-6^−^ hosts may permit one to determine whether mIL-6 production in the tumor microenvironment is as important for vIL-6-driven plasmablastic tumors as it is for Myc-driven plasma cell tumors.^13^ To facilitate this research and enhance our understanding of vIL-6-promoted B-cell disorders, strain C.vIL6 has been made available to the scientific community via the Jackson Laboratory (Bar Harbor, ME, USA) sperm cryopreservation and strain recovery program.

## Figures and Tables

**Figure 1:**
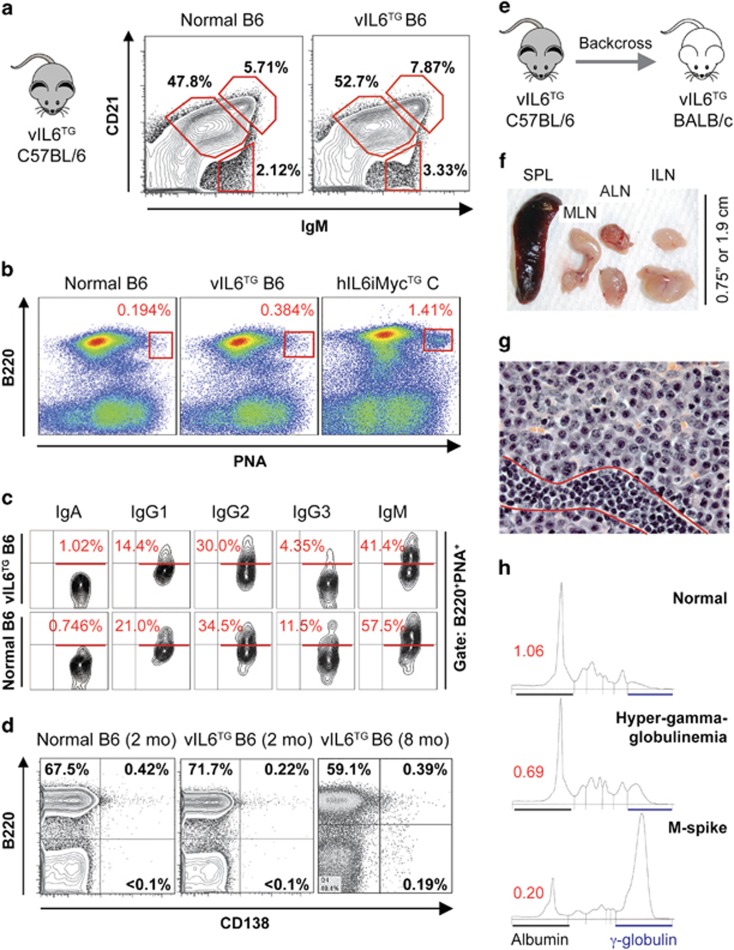
B6-to-C transfer of transgenic vIL-6 expression exacerbates MCD-like disease in mice. (**a**) Splenic B-cell compartments in vIL6-TG B6 mice are normal. Splenocytes were gated on B220 (CD45) and labeled with antibodies to CD21 and IgM to enumerate follicular (CD21^+^ IgM^lo^), transitional (CD21^−^ IgM^hi^) and marginal zone (CD21^hi^ IgM^hi^) B cells. The frequency of these cells was comparable in age-matched vIL6-TG and normal mice. (**b**) Normal numbers of spontaneous germinal center (GC) B cells in vIL6-TG B6 mice. Splenocytes were labeled with antibodies to B220 and PNA (peanut agglutinin) to enumerate GC B cells (red rectangles). The frequency of these cells was comparably low in vIL6-TG and normal B6 mice, but elevated in C mice that harbored a human IL6 and mouse Myc transgene (hIL6iMyc).^14^ The latter were included as positive control. (**c**) Normal isotype profile of GC B cells in vIL6-TG B6 mice. Splenocytes, gated on B220 and PNA, were labeled with antibodies to the indicated isotypes (immunoglobulin heavy-chains) expressed on the cell surface. Percentages of cells that express a given isotype are indicated in red. Shown is a representative result from independent measurements, using different, age-matched transgenic and normal mice. The immunoglobulin expression pattern was comparable in the two strains of mice. (**d**) Low numbers of splenic plasmablasts (PBs) and plasma cells (PCs) in vIL6-TG B6 mice. Forward scatter- and side scatter-gated splenocytes were evaluated for the expression of B220 and CD138 (syndecan-1) to distinguish B220^+^CD138^−^ B cells and B220^−^CD138^+^ PCs. Normal mice (used as control, left panel) and age-matched vIL6-TG mice (center) contained <0.1% PCs. Eight-month-old transgenic mice (right) exhibited a small but reproducible increase in PCs that can be attributed to aging. (**e**) Generation of C.vIL6 congenic mice. The vIL6 transgene was transferred from B6 to C by 10 generations of introgressive backcrossing. (**f**) Splenolymphadenopathy due to plasmablastic MCD-like disease in C.vIL-6 mice. Shown is a photomicrograph of the enlarged spleen (~350 mg, left), mesenteric lymph node (MLN) and left and right axillary (ALN) and inguinal (ILN) lymph nodes from a diseased mouse. (**g**) Histopathologic examination of one of the ILNs shown in **f** reveals an abnormal accumulation of aberrant PCs in medullary cords. A streak of small lymphocytes located at the bottom (indicated by two red lines) is included for size comparison (H&E, 63 ×). (**h**) Elevation of serum Ig levels in 215-day-old C.vIL-6 mice. Shown are densitographic representations of the abundance of serum proteins fractionated by electrophoresis. Albumins and γ-globulins are indicated by black and blue horizontal lines, respectively. A mouse exhibiting hyper-gammaglobulinemia (center) or M-spike (bottom) is compared with an age-matched normal C mouse (top). The ratios of albumin to γ-globulin are indicated by red numbers. H&E, hematoxylin and eosin.

**Figure 2:**
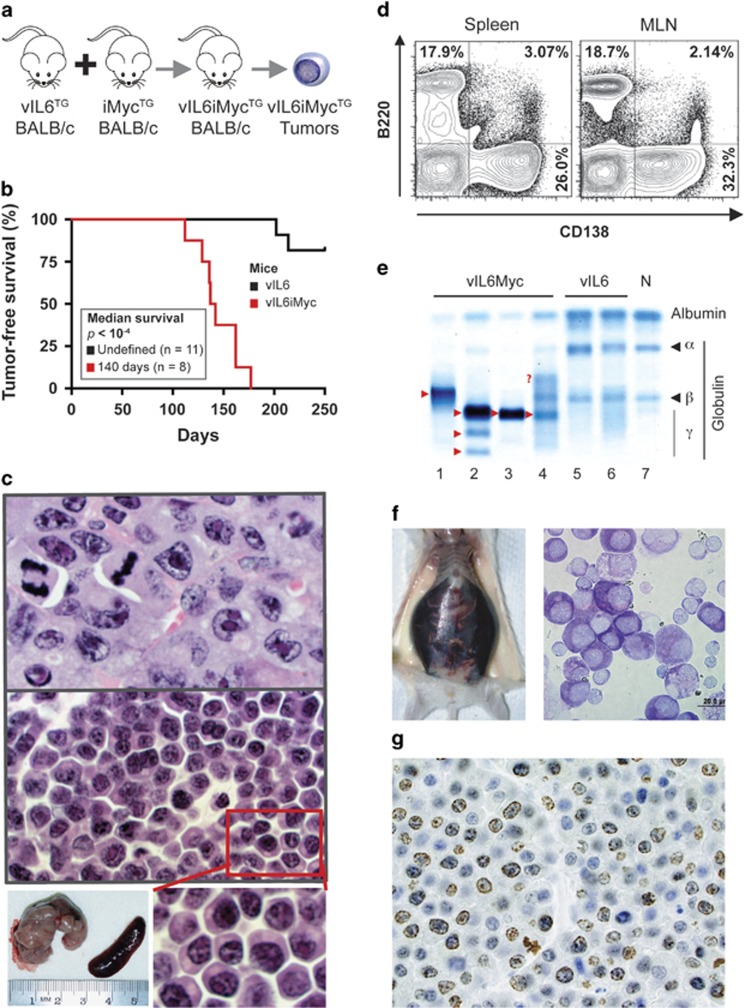
Double-TG C.vIL6iMyc mice are prone to malignant B-cell tumors associated with KSHV in humans. (**a**) Schematic overview of generating C.vIL6iMyc-TG mice and observing them for tumor development. Heterozygous TG vIL6 mice were intercrossed with homozygous-TG iMyc mice. Double-TG vIL6iMyc mice, which occurred at the expected frequency of ~50%, were identified by PCR-based genotyping. The mice were left untreated and observed for tumor manifestation. Neoplastic tissues were analyzed using flow cytometric and histopathological methods. (**b**) Line graphs indicating tumor-free survival of single-TG vIL6 mice (black, *n*=11) and double-TG vIL6iMyc mice (red, *n*=8). Median tumor-free survival of vIL6 mice (undefined at 250 days of age) was significantly longer than that of vIL6iMyc mice (140 days), using Mantel–Cox log-rank analysis (*P*<10^−4^). Single TG C.iMyc mice are not included in the graph because spontaneous tumors are rare and tumor-free survival has not been determined yet. In a previous study, we found that 8 of 76 (10.5%) mice had developed tumors by 410 days of age (median: 251 days; mean: 269±64.3 days; range: 213–410 days).^14^ Thus, although definitive data are not available at this juncture, C.iMyc mice appear to be even less prone to malignancy than C.vIL6Myc mice are. (**c**) Representative H&E-stained tissue section of a plasmablastic neoplasm (top, original magnification × 100) and plasma cell proliferation (center, original magnification × 100). Both lesions were obtained from the same C.vIL6iMyc mouse. The plasmablastic neoplasm showed multi-organ involvement including bone marrow, enlarged lymph nodes, liver, spleen and full thickness small bowel tumors. The plasma cell proliferation was observed in focal areas of mesenteric fat and peri-nephric tissue. The greatly enlarged mesenteric lymph node, which was effaced by the plasmablastic neoplasm, is shown at the bottom left. (**d**) Flow cytometric histograms of a representative plasmablastic neoplasm. The tumor arose in a vIL6iMyc mouse at 130 days of age. Forward scatter- and side scatter-gated cells from spleen (left) or mesenteric lymph node (MLN, right) were evaluated for the expression of B220 (B-cell marker) and CD138 (syndecan-1, plasma cell marker). Nearly one-third of cells in both tissues were B220-CD138+ plasma cells. (**e**) Serum protein electropherograms of four different tumor-bearing C.vIL6iMyc mice (lanes 1–4), two C.vIL6 mice with incipient MCD-like disease (lanes 5–6) and one age-matched normal (N) control (lane 7). M-spikes (paraproteins) are indicated by red arrowheads pointing right. The red question mark denotes a weak additional extragradient in lane 4. (**f**) PEL-like neoplasia in a C.vIL6iMyc mouse. Presented to the left is a photograph of a tumor-bearing mouse containing ~4 ml of hemorrhagic ascites. The Wright–Giemsa-stained cytofuge specimen to the right (bar, 20 μm) includes a cluster of tumor cells that consists mainly of malignant plasmablasts and plasma cells. The cells are large, basophilic and usually contain one eccentric nucleus. Bi-nucleated cells occur occasionally. (**g**) Ki67 immunostain of plasmablastic neoplasm in a C.vIL6iMyc mouse. Proliferation index is 70–75%. Note that this neoplasm and the tumors displayed in **c** and **f** share unifying features of human KSHV-associated PBL, PBM and PEL. These include a very aggressive clinical course and morphology of diffuse, cohesive proliferations of cells that resemble immunoblasts with varying degrees of plasmacytoid differentiation and frequent mitotic figures. Because the diagnostic distinction of the human disease entities depends in large measure on the broader clinical-pathologic context, for example, site of disease involvement, presence of immunodeficiency and infection with KSHV,^15^ it is difficult if not impossible to identify accurate counterparts of these entities in the mouse model. H&E, hematoxylin and eosin.
