# Supplementary material for: KSHV-encoded vIL-6 collaborates with deregulated c-Myc to drive plasmablastic neoplasms in mice
Source: Blood Cancer J. 2016 Feb 26;6(2):e398–. doi: 10.1038/bcj.2016.6 (PMC4771969; doi:10.1038/bcj.2016.6)
Supplement: Supplementary Information [file bcj20166x1.docx]

**Supplemental Methods**

**Mice**

All studies were performed on the BALB/c (C) background and approved under IACUC Protocol 0006A56361. The generation of vIL6-TG B6 mice has been described elsewhere [^1^](#_ENREF_1). The generation of C.iMycΔEμ “knock-in” mice has also been reported in a previous paper [^2^](#_ENREF_2). Strain C.vIL6; i.e., the H2-K-driven vIL6 transgene on the C background described in this paper, has been made available to the scientific community via the JAX (Jackson Laboratory, Bar Harbor, Maine) sperm cryopreservation and strain recovery program. Strain ID number is 911198. With a demonstrated success rate of 91% (reported by JAX personnel), C.vIL6 mice should be readily recovered and shipped worldwide (upon request) to other investigators.

**Diagnosis and histopathology of plasmablastic neoplasms**

Incipient tumors were detected by monitoring mice for health status parameters, including occurrence of hind limb weakness or paralysis. The diagnosis of plasmablastic neoplasia was established at necropsy of tumor-bearing mice and confirmed histologically using criteria described in the Bethesda classification of mouse hematopoietic tumors [^3^](#_ENREF_3). Four-μicrometer sections of paraffin-embedded tissues were stained with hematoxylin and eosin (H&E) and evaluated by a board-certified human hematopathologist (Carol Holman) experienced with B-lineage blood cancers in laboratory mice.

**Flow-cytometric analysis of plasmablastic neoplasms**

Antibodies to B220 (6B2) and CD138 (281-2) were purchased from eBioscience (San Diego, CA) and BD Biosciences (San Jose, CA), respectively. To obtain single cell suspensions of lymphocytes, lymphoid tissues were harvested and minced between frosted glass slides. ACK lysis (Lonza, Radnor, PA) was used to remove red blood cells. For flow analysis, one million cells were washed and re-suspended in staining buffer that consisted of balanced salt solution, 5% bovine calf serum and 0.1% sodium azide. Non-specific binding of antibody was blocked using 10 μl rat serum (Jackson Immunoresearch, West Grove, PA) and 10 μg 2.4G2 (BioXCell, West Lebanon, NH). Cells were labeled on wet ice in the dark. Samples were run on a FACSCANTO II (Becton Dickinson, San Jose, CA) and data were analyzed using FlowJo (Tree Star, Ashland, OR).

**Detection and isotyping of serum paraproteins**

Whole blood was collected from mice at necropsy, using heart puncture. Blood was transferred to EDTA-coated Microtainer tubes (Becton Dickinson) and spun for 5 min at 14,000 RPM to obtain serum. After centrifugation, serum was removed and frozen until the time of analysis. Serum protein elecrophoresis was used to detect paraproteins (M-spikes) and polyclonal elevations of Ig (hyper-gammaglobulinemia). Serum proteins were fractionated on Hydragel Protein(e) K20 gels using a Sebia elecrophoresis chamber (90 V constant; 40 min migration time; 12 ± 3 mA). Paraproteins were isotyped using the Mouse Immunoglobulin Isotyping ELISA from BD Pharmingen according to the manufacturer’s recommendations. Modifications included the dilution of the serum samples and the HRP-labeled secondary antibody in blocking buffer that contained 0.05% Tween 20. Addidtionally, the HRP-labeled antibody was used at a titer of 1:200. ELISA 96-well microplates were read at 450 nm using the Multiskan Spectrum from Thermo Scientific.**Supplemental Figure Legends**

**Supplemental Figure 1: Histopathologic features of MCD-like disease in C.vIL6 mice.**

**(a)** Immunofluorescence analysis of spleen showing abundance of IgM^High^ plasmablasts and plasma cells in inter-follicular areas (top and center panels) after labeling with antibody to FITC-conjugated IgM. B-cell follicles were visualized with the help of FITC-labeled antibody to B220 (bottom panel). The approximate borderlines between follicular and inter-follicular areas (IFA) are indicated by red lines, which have been drawn using the Adobe Illustrator Pencil tool. Note that B-cell follicles, which appear as black “punched out” areas in the top and center panels, actually contain an abundance of IgM^Low^ B cells that are not visible due to image thresholding.

**(b)** Increased numbers of megakaryocytes in the spleen (top panel) and bone marrow (bottom) of vIL6-TG mice. Shown are representative areas containing an abundance of megakaryocytes (indicated by colored arrowheads pointing right), which were repeatedly noticed during histopathologic examination of tissue sections. No attempt was made to statistically validate the findings using quantitative histomorphometric methods.

**(c)** Aggregates of atypical plasmablasts and plasma cells in unusual tissue sites. Left panel shows a small cluster of hyperchromatic plasmablasts (indicated by red box) in a periportal field of liver. Right panel presents a more extensive infiltrate of plasmablasts and plasma cells (red arrowheads) in the pericapsular region of a lymph node. The lymph node capsule (black asterisk) and a sliver of the node’s cortical region are shown to the upper left. The black asterisk denotes loose connective tissue surrounding the lymph node capsule.

**Supplemental Figure 2: Elevation of serum Ig levels and occurrence of M-spikes in vIL6-TG mice.**

**(a)** Serum M-spikes and polyclonal elevations of serum Ig in 4 different C.vIL6 mice 209-390 days of age (left panel) and 4 different C.vIL6iMyc mice younger than 180 days of age (right panel). Shown are densitograms of serum protein fractions separated electrophoretically. ID numbers and age of mice are indicated. Also included are numerical values of albumin-to γ-globulin ratios, ranging from 1.16 in the control (top left) to 0.16 in a C.vIL6iMyc mouse. The C.vIL6 samples represent the full range of changes indicating stages of Ig^+^ plasma cell tumor progression: normal Ig levels (#1482) that transition to moderate hyper-γ-globulinemia (#1478), which progresses to multiple small M-spikes on a background of pronounced hyper-γ-globulinemia (#1400) and culminates in a distinct M-spike (#1477). Four of 4 C.vIL6iMyc sera contained M-spikes, in case of #1320 probably representing 2 independent Ig-producing cell clones.

**(b)** Serum M-spikes of 4 mice, included in panel a, were isotyped using ELISA. Serum samples were logarithmically diluted, from 10^-4^ to 10^-8^, and probed with antibodies to Ig heavy-chains and light-chains. This is indicated vertically to the left of the photographic images of the two 96-well micro-plates. The 2 rightmost columns of the plates were used for positive and negative controls (Co) supplied by the manufacturer. Major and minor heavy and light chain M spikes, based on ELISA signal strength, are indicated by red and blue circles, respectively. M-spikes in sera that contained single paraproteins were readily determined: IgAκ in case of 1292 and IgG2bκ in case of 1287. The latter may also contain a smaller IgG3κ spike. The bi-clonal densitogram of mouse 1320 (see panel a) was attributed to IgG1κ and IgG2bκ producing cell clones. Sample 1400 was difficult to isotype, as one might have expected from the result presented in panel a. Nonetheless, a major IgAκ spike was distinguished from two somewhat less abundant γ1 and γ2b spikes. Additional (minor) spikes may exist.

**Supplemental References**

1. Suthaus J, Stuhlmann-Laeisz C, Tompkins VS, Rosean TR, Klapper W, Tosato G*, et al.* HHV-8-encoded viral IL-6 collaborates with mouse IL-6 in the development of multicentric Castleman disease in mice. *Blood* 2012 May 31; **119**(22)**:** 5173-5181.

2. Duncan K, Rosean TR, Tompkins VS, Olivier A, Sompallae R, Zhan F*, et al.* (18)F-FDG-PET/CT imaging in an IL-6- and MYC-driven mouse model of human multiple myeloma affords objective evaluation of plasma cell tumor progression and therapeutic response to the proteasome inhibitor ixazomib. *Blood Cancer J* 2013; **3:** e165.

3. Morse HC, 3rd, Anver MR, Fredrickson TN, Haines DC, Harris AW, Harris NL*, et al.* Bethesda proposals for classification of lymphoid neoplasms in mice. *Blood* 2002 Jul 1; **100**(1)**:** 246-258.
