# Supplementary figures and images for: KSHV-encoded vIL-6 collaborates with deregulated c-Myc to drive plasmablastic neoplasms in mice
Source: Blood Cancer J. 2016 Feb 26;6(2):e398–. doi: 10.1038/bcj.2016.6 (PMC4771969; doi:10.1038/bcj.2016.6)

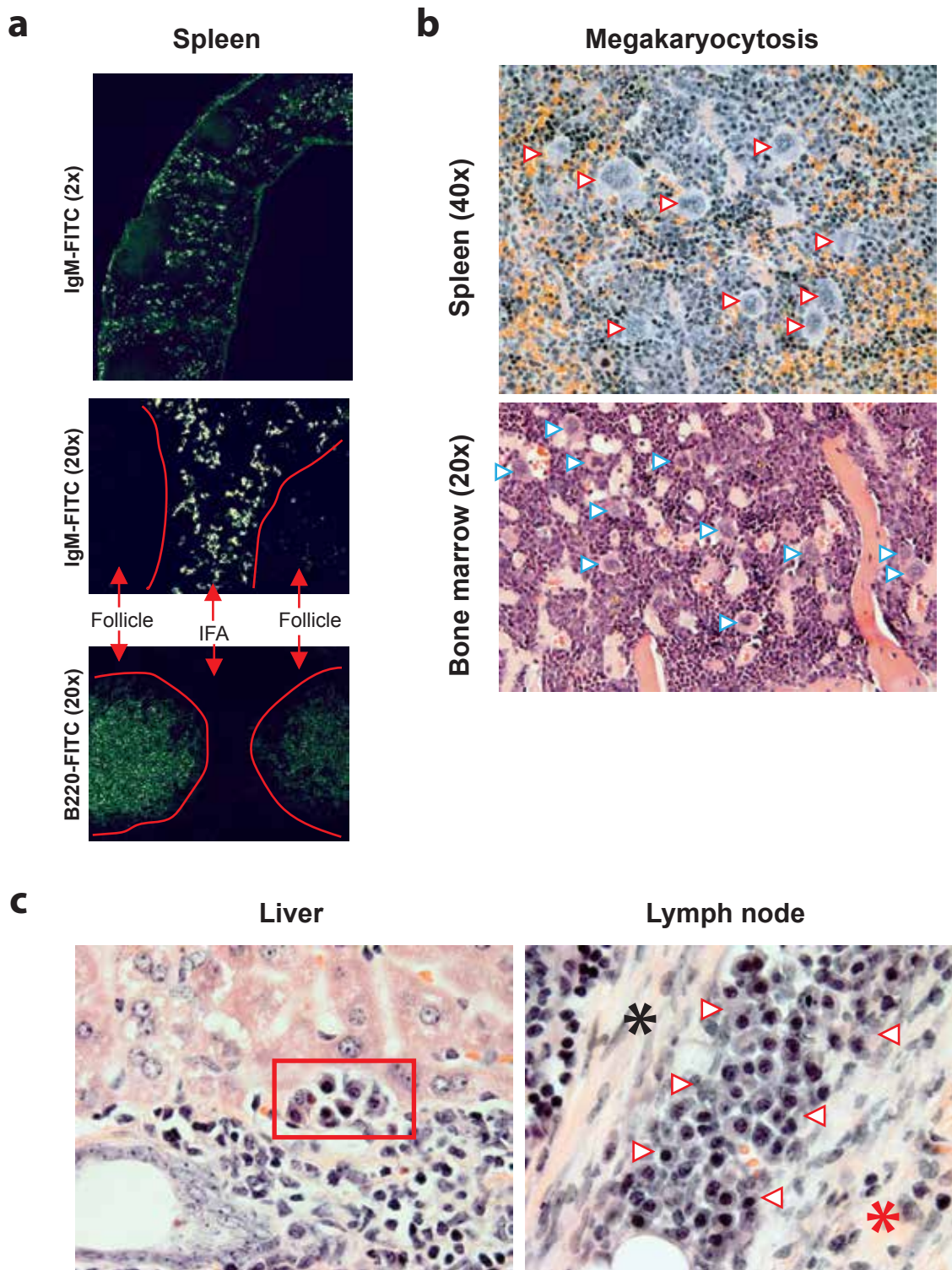

Supplemental Figure 1: Rosean *et al.*

Supplement: Supplementary Figure 1 [file bcj20166x2.pdf]

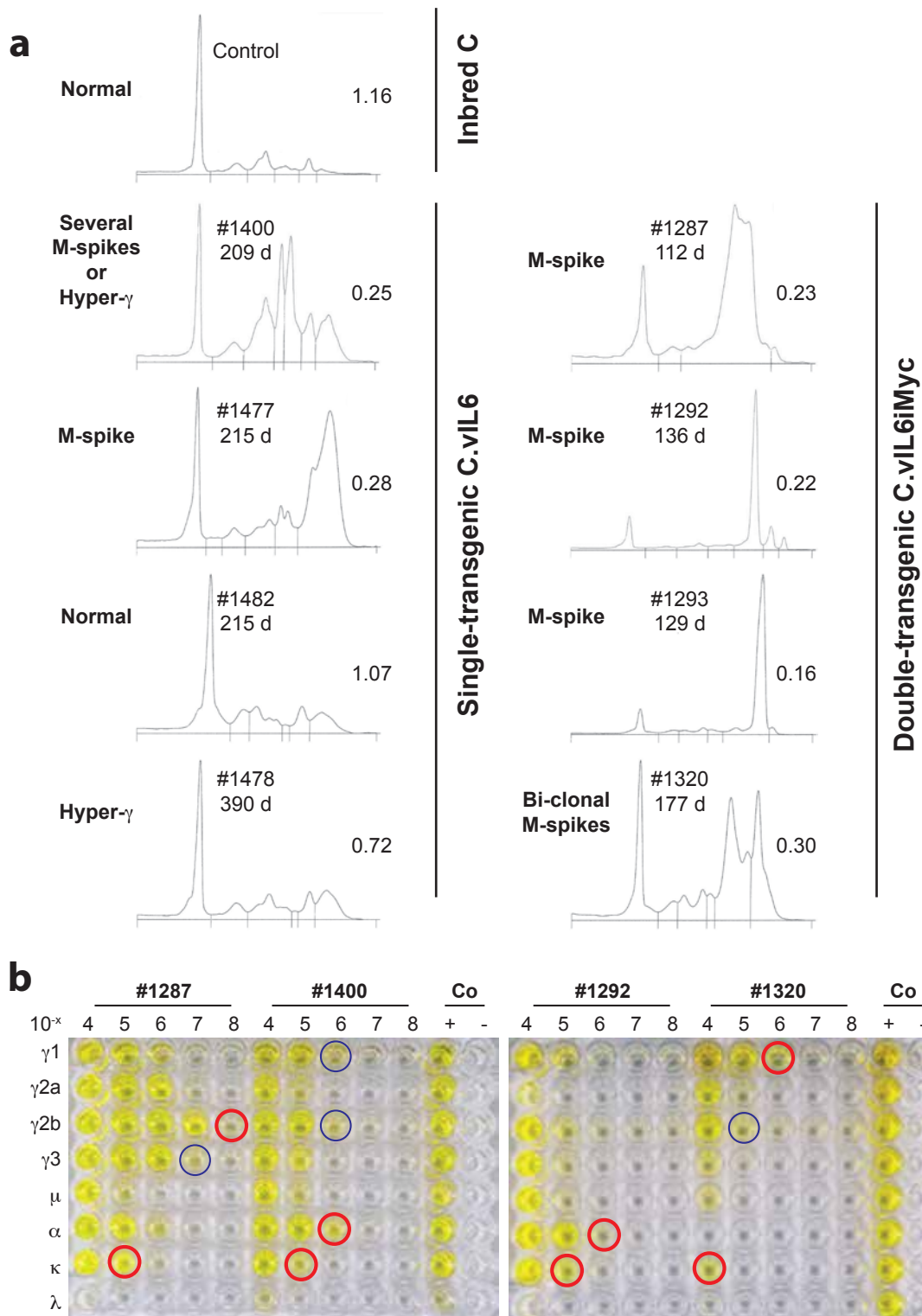

Supplemental Figure 2: Rosean *et al.*

Supplement: Supplementary Figure 2 [file bcj20166x3.pdf]
